# Supplementary material for: A study protocol for the assessment of antibiotic use and bacterial antimicrobial resistance among children under five years of age: Implications for a resource-limited setting
Source: PLoS One. 2025 Nov 19;20(11):e0330402. doi: 10.1371/journal.pone.0330402 (PMC12629471; doi:10.1371/journal.pone.0330402)
Supplement: Attachment — Submitted filename: Responses to reviewers comments 2.docx [file pone.0330402.s002.docx]

Dr. Maidelyn R. Peregrin
PLOS ONE

**Response to editorial and reviewers comments**

1. Thank you for providing this information regarding Figure 1. Can you confirm what data you used and whether base map outlines were used? As ArcGIS belongs to ESRI, the correct attributions will need to be given. I've pasted the information from ESRI below:

Authors must have ESRI permission to use sample data. Any map or other creation by the author using ESRI software belongs to the author and can be included in the paper. Base map outlines are OK as long as there's proper attribution to ESRI. Required statements are OK if ESRI gave permissions and requires those statements: "“Copyright © XXX. All rights reserved” or “Esri reserves the right to grant permission for any other use of the Image”.

For more information on our Licenses and Copyright policy please head to our website, linked here: <https://journals.plos.org/plosone/s/licenses-and-copyright>

**Authors response:** The map was created entirely with our own dataset on top of an ESRI base map. No ESRI sample data or other third-party datasets were used. To avoid any questions, we included the full ESRI attribution line, not just the base map credit, so all sources are clearly covered:

*“Sources: Esri, TomTom, Garmin, FAO, NOAA, USGS, © OpenStreetMap contributors, and the GIS User Community, ESRI, USGS.”* Please check out the update map now in the manuscript.

2. We note that you state the following regarding your Data Availability: "In addition to what is already captured under data availability, from 528-536, we included a whole section on dissemination plan or data sharing plan which reads - Dissemination plan
The findings of this work will be disseminated to healthcare professionals and AMR stakeholders in Sierra Leone, such as the technical working group on AMR and regulatory bodies to inform enforcement strategies and interventions to control irrational antibiotic use. We will also disseminate our findings in academic and professional meetings. Manuscripts will be developed and sent to peer-reviewed journals for publication. In addition, abstracts will be submitted for presentations at local and international scientific and professional meetings. The results of this project will provide evidence-based data to inform the development of policies, guidance, strategies, and interventions to promote antibiotic stewardship in Sierra Leone. "

We note that your Data Availability statement states the following: "The protocol and all data generated will be available from the corresponding authors upon request and will be deposited in the College of Medicine and Allied Health Sciences, University of Sierra Leone data repository. Deidentified research data will be made publicly available when the study is completed and published."

Please note that PLOS does not allow authors to be the sole contact for data inquiries. At this time, please confirm that we may update your Data Availability statement to the following: "The protocol and all deidentified data generated will be made publicly available when the study is completed and published.

**Authors response:** Yes. You may update the Data Availability statement.

Yours Sincerely,

Onome T. Abiri
